# Role of Acute Physiotherapy in Bilateral Medial Medullary Infarction: A Case Report

**DOI:** 10.7759/cureus.62828

**Published:** 2024-06-21

**Authors:** Takahiro Miyashita, Eiki Tsushima, Kouhei Nagamine

**Affiliations:** 1 Department of Physical Therapy, Saku Central Hospital, Saku, JPN; 2 Health Sciences, Hirosaki University, Hirosaki, JPN; 3 Department of Rehabilitation Medicine, Shinshu University School of Medicine, Matsumoto, JPN; 4 Department of Rehabilitation, Saku Central Hospital, Saku, JPN

**Keywords:** respiratory care, respiratory disorders, dysphagia, quadriplegia, bilateral medial medullary infarction

## Abstract

Bilateral medial medullary infarction (BMMI) is a rare stroke syndrome, which frequently has poor clinical outcomes. Reports on physical therapy for BMMI are few because of its poor prognosis. Therefore, this report aims to present a patient who developed BMMI and underwent well-considered rehabilitation. A 67-year-old man presented to our clinic with an acute onset of vomiting and dizziness. Magnetic resonance imaging (MRI) showed no abnormal signal intensity, and the patient was admitted for peripheral dizziness. On day two, he developed quadriplegia, bulbar palsy, and respiratory impairment, such as prolonged apnea. A second MRI revealed a high-intensity lesion in the bilateral medial medulla oblongata. He was diagnosed with BMMI, and rehabilitation treatment was initiated. On day 16, his sputum volume increased, and he could not expectorate effectively due to decreased coughing ability. Therefore, mechanical insufflation-exsufflation (MI-E) was performed to improve his airway clearance. On day 21, he developed aspiration pneumonia (AP), which became severe and led to acute respiratory failure. Nasal airway intubation and oxygen flow of 5 L/minute were initiated. His respiratory function was not seriously aggravated, and recurrent AP was prevented with the application of respiratory physiotherapy procedures, such as postural drainage, in collaboration with other medical staff, and MI-E. On day 60, the patient was transferred to the recovery phase rehabilitation ward. BMMI tends to worsen swallowing disorders progressively and is associated with a high risk of severe AP. Providing physiotherapy in the acute phase is important to reduce the risk of serious illness.

## Introduction

Medial medullary infarction is a rare type of stroke, accounting for approximately 0.52%-1.5% of all cerebral infarctions and 21.6%-29.3% of all medullary infarctions [1-4], making it rarely encountered in physical therapy practice. The ventral medial vascular territory of the medulla oblongata includes the pyramidal tract, medial colliculus, and hypoglossal nerve nucleus and is known to present with various symptoms. Medial medullary nutrient vessels are the anterior spinal arteries, and when these arteries are occluded, they can cause bilateral medial medullary infarction (BMMI), which can be severe. BMMI usually presents with quadriplegia and respiratory failure. Dysphagia may occur, and pneumonia is fatal in 23.8% of patients [5].

Although some studies have investigated the clinical features and outcomes of BMMI [6,7], few have investigated the outcomes of severe cases, and limited information is available on the type of physical therapy that should be provided during the acute phase. Therefore, this report aims to describe the role of physiotherapy in the acute phase of BMMI.

## Case presentation

Patient information

**Figure 1:**
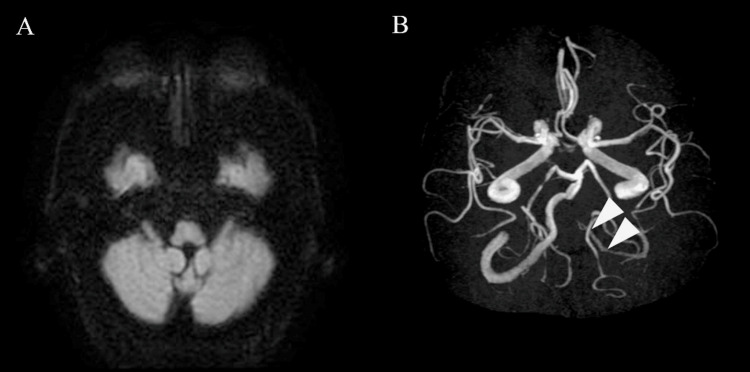
Head MRI findings on the day of admission. A: New high signals were not noticeable on DWI. B: No left vertebral artery delineation on MRA. MRI: magnetic resonance imaging; DWI: diffusion-weighted imaging; MRA: magnetic resonance angiography

A 67-year-old man presented to our emergency department with a chief complaint of experiencing multiple episodes of vomiting that morning and dizziness. His height was 168 cm, weight was 71.5 kg, and body mass index was 25.3 kg/m^2^. He had a history of hypertension, diabetes mellitus, and cerebral infarction. No physical function disabilities associated with pre-existing medical conditions were observed. Head computed tomography showed no new hemorrhage, and diffusion-weighted imaging (DWI) of head magnetic resonance imaging (MRI) showed no high signal (Figure 1A). Magnetic resonance angiography (MRA) did not depict the left vertebral artery (Figure 1B).

**Figure 2:**
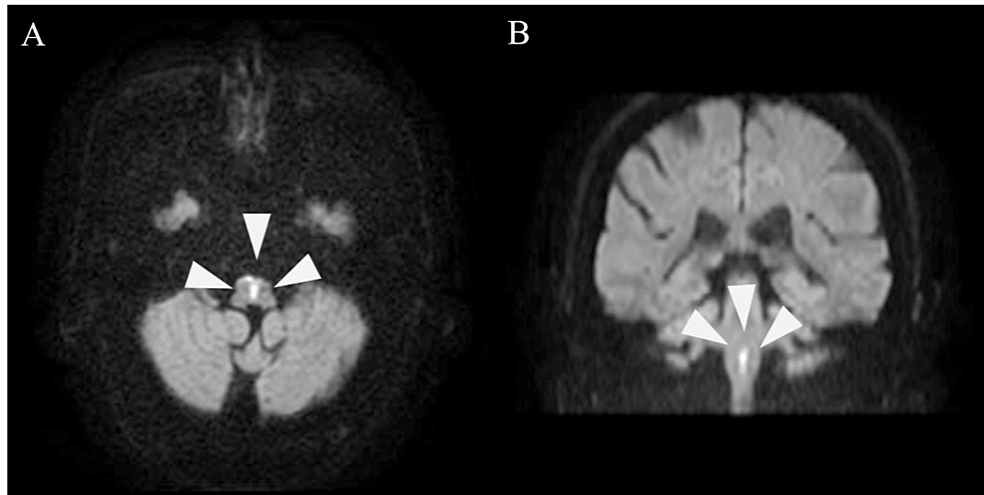
Head MRI findings on the day after admission. A: Axial DWI view. B: Coronal DWI view. High signal in the bilateral medial medullary in both A and B. MRI: magnetic resonance imaging; DWI: diffusion-weighted imaging

Based on other examinations, the patient was admitted for observation with a diagnosis of peripheral vertigo. On the morning of the second day of admission, the patient experienced severe quadriplegia and dysarthria. Emergency head MRI revealed a high DWI signal from the ventral to dorsal medial medulla oblongata bilaterally (Figures 2A, 2B).

The findings were consistent with the heart sign [8], a characteristic feature of BMMI, and he was diagnosed with BMMI. Biochemical and hematological examination revealed that white blood cell counts, neutrophils, C-reactive protein levels, HbA1c levels, and blood glucose and urinary glucose levels all exceeded their reference values (Table 1). Rehabilitation with physiotherapy, occupational therapy, and speech therapy was initiated two days after admission.

**Table 1:** Biochemical and hematological examination on admission. WBC: white blood cell; CRP: C-reactive protein; HbA1c: glycosylated hemoglobin

Parameter	Result	Reference range
WBC	9,900/μL	4,000–9,000/µL
Neutrophils	78.6%	37-73%
CRP	10.06 mg/dL	<0.3 mg/dL
D-dimer	3.4 μg/mL	<1.0 μg/mL
HbA1c	6.5%	4.6–6.2%
Glucose	177 mg/dL	75–109 mg/dL
Urinary sugar	4+ (qualitative)	-

Clinical findings

The consciousness level was assessed using the Glasgow Coma Scale (GCS). The GCS score was 13, with an eye-opening score of 3, a verbal response score of 4, and a motor response score of 6. The patient’s blood pressure was 172/106 mmHg, heart rate was 71 beats/minute, saturation of percutaneous oxygen (SpO_2_) was 92%-95%, body temperature was 37.1°C, and respiratory rate was 17 breaths/minute. The respiratory pattern was Cheyne-Stokes breathing with a strong snoring sound and apnea for approximately 15 seconds. The patient was instructed to cough but could not do so. He developed slurred speech, with only occasional intelligible words. The swallowing function was assessed using the Functional Oral Intake Scale (FOIS). The FOIS score was 1. The patient had difficulty synchronizing breathing and swallowing, with inhalation occurring immediately after swallowing. Furthermore, saliva caused aspiration. Motor paralysis was assessed using the Brunnstrom recovery stage (BRS) and Stroke Impairment Assessment Set (SIAS). The BRS (right/left) was III/III in the upper limbs, III/IV in the hands, and III/III in the lower limbs. The SIAS score was 25/76, and the right side was evaluated as paralyzed. Sensory examination revealed mild insensitivity to superficial sensation and moderate insensitivity to deep sensation on the right side of the body and no abnormalities on the left side. Manual muscle testing (right/left) revealed biceps graded at 2/3, triceps at 1/3, iliopsoas at 1/2, quadriceps at 1/4, hamstrings at 1/2, and tibialis anterior at 1/1.

Physiotherapeutic plan

The patient had severe tetraplegia and sensory loss on the right side of his body. Thus, the primary aim was to prevent pressure ulcers, atelectasis, deep vein thrombosis, and contractures. The secondary aim was to prevent aspiration pneumonia (AP) caused by aspiration due to delayed swallowing reflexes and airway secretion retention due to coughing difficulties. The physiotherapy program was planned to include joint range of motion training of the neck, trunk, and extremities to prevent contractures and automatic assistance exercises for the left upper and lower limbs. Additionally, supine and lateral positioning were planned to prevent pressure sores. Oral care was also planned to prevent AP. A moisturizing gel was used to keep the mouth moist, and dry mucus was removed with a sponge brush. Antigravity activities were planned based on all activities requiring voluntary muscle use, such as turning, getting up, sitting, standing, and walking, to prevent deep vein thrombosis and encourage using paralyzed muscles.

Physiotherapeutic intervention

**Figure 3:**
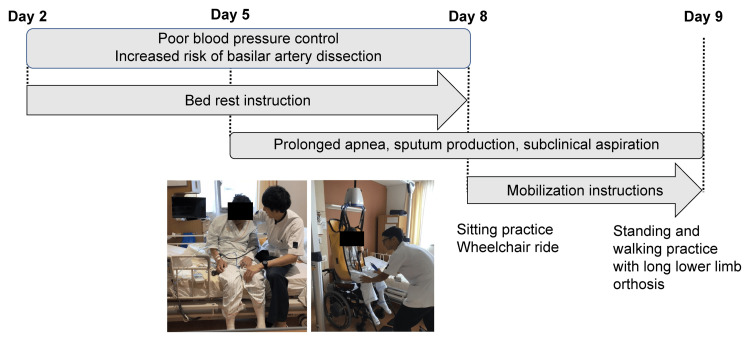
Physiotherapy progress in the first part. Image credits: Takahiro Miyashita.

The patient was instructed by his physician to remain in bed because of the high risk of enlargement of the divergent aneurysm. Resting joint range of motion exercises for the neck, trunk, and extremities were initiated with a systolic blood pressure of 160 mmHg as the upper limit. On day three of admission, tetraplegia worsened, and the patient’s BRS was II/III in the upper limbs, II/IV in the hands, and II/III in the lower limbs. On day five, the patient was started with a head-up angle of 15° and checked for 15-minute blood pressure fluctuations. On the same day, the systolic blood pressure fluctuated by >40 mmHg at a 30° head-up position. Therefore, we tried to gradually increase the head-up over several days. On day eight, the patient’s condition began to stabilize, and he was transferred to a reclining wheelchair and kept in the end-occupant position. The head and neck were difficult to hold in the end-sitting position. Therefore, they were held in the midline position with the therapist’s assistance. Additionally, neck extension holding exercises were performed. Cervical extension holding exercises were also performed in the end-sitting position, grasping the occipital ridge and mandible until the patient complained of fatigue. Standing holding exercises using bilateral long-limb orthoses and walking exercises with full rearward assistance were initiated on day nine. Standing hold exercises were performed for 10 sets of one minute and walking exercises for two to five sets of 10 minutes on the ward based on patient fatigue. The patient gradually had more opportunities to leave the bed (Figure 3).

**Figure 4:**
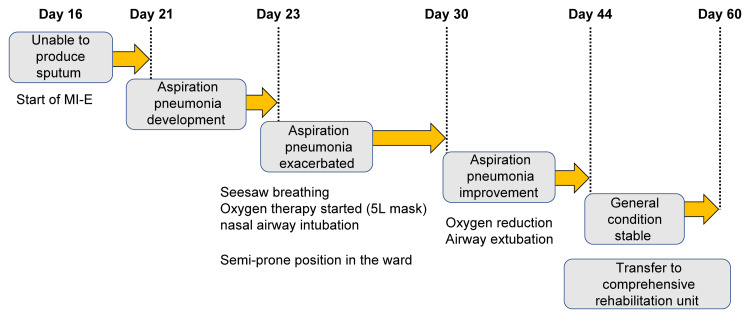
Physiotherapy progress in the second part. Image credits: Takahiro Miyashita.

However, his respiratory condition remained unstable, and the sputum amount increased from day 16. Because his cough was weak and he could not expectorate sputum, a mechanical insufflation-exsufflation (MI-E) device was used to improve airway clearance. The patient could not expectorate sufficiently using the normal automatic mode setting. Therefore, the cuff-track mode was used to lower the inspiratory pressure below the expiratory pressure to promote expectoration, following previous studies [9]. However, AP developed early in the morning of day 21, and his respiratory condition deteriorated. Therefore, a 5 L oxygen mask and nasal airway were used. Mobilization was temporarily stopped, and an approach was used to improve airway clearance for respiratory impairment. MI-E was continued and performed at every intervention. Additionally, positional drainage was performed in the semi-prone position by the ward nurse every two hours to facilitate dorsal thoracic release and airway secretion migration. During physiotherapy, the patient was placed in the semi-prone position, the anterior and dorsal thorax were grasped, and chest compression with exhalation was performed for 20 minutes on each side. The sputum amount decreased, the nasal airway was removed on day 30, and MI-E was completed. On day 37, the patient resumed using the reclining wheelchair. On day 56, respiratory symptoms stabilized with a notable improvement in AP; thus, oxygen therapy was ceased. The patient was transferred to the rehabilitation ward on day 60 (Figure 4). The initial and final assessment of this case is summarized in Table 2.

**Table 2:** Outcome measures. BRS: Brunnstrom recovery stage; SIAS: Stroke Impairment Assessment Set; MMT: manual muscle testing; FOIS: Functional Oral Intake Scale

Outcome measures	At admission (on hospital day 3)	On transfer (on hospital day 60)
Respiratory state	Apnea + tongue root subsidence	Chain stokes breathing; apnea persists
BRS (Rt/Lt)	Upper limb (II/III); hand (II/IV); lower limb (II/III)	Unchanged
SIAS (points)	25/76	27/76
Sensation	Superficial sensation is mildly blunted; deep sensation is moderately dull	Unchanged
MMT (Rt/Lt)	Iliopsoas: 1/2; quadriceps: 1/4; biceps femoris: 1/2; tibialis anterior: 1/1	Iliopsoas: 1/1; quadriceps: 2/4; biceps femoris: 1/3; tibialis anterior: 2/2
FOIS (level)	1	3

## Discussion

The patient developed obvious symptoms of neurological dropout after admission. A retested head MRI revealed BMMI. The dissecting basilar artery aneurysm extended to the confluence of the vertebrobasilar arteries, and the anterior spinal artery, a branch of the vertebrobasilar artery, was considered to have developed blood flow failure, leading to ischemia in the medial medulla oblongata on both sides. Cerebral infarction in the vertebrobasilar artery region may not show a high DWI signal on a head MRI taken within 48 hours of onset [10]. Therefore, attention should be paid even if there is no obvious infarcted area on imaging in the early stage of onset.

Clinical manifestations of BMMI include motor dysfunction, nystagmus, dysarthria, dysesthesia, sublingual nerve palsy, respiratory failure, and dysphagia [5]. The lateral medulla oblongata contains the nucleus tractus solitarius, nucleus suspectus, and reticular formation related to swallowing function and respiratory neurons and pre-Bötzinger complex related to respiratory function [11]. These nerve nuclei are anatomically adjacent to the medial medulla oblongata, and extensive ischemia in the medial medulla oblongata can cause dysphagia and respiratory impairment [12]. Therefore, BMMI with respiratory failure and dysphagia should be considered to represent a severe stroke.

In this case, the most problematic aspect was prolonged respiratory disturbance. Neurological symptom exacerbation associated with cerebral artery dissection has been reported to occur within three days [13]. However, Jalal and Menon reported an association between BMMI and respiratory failure and a certain delay between the onset of neurological symptoms and the development of respiratory problems [14]. Furthermore, AP is a major cause of death in patients with BMMI, and respiratory failure can be fatal if airway management is inadequate [15]. In our case, manually assisted coughing was ineffective in draining airway secretions. Although MI-E was used on day 16 and expectoration was observed, AP developed on day 21. Regarding the course of AP in the early morning, the disease may develop because of subclinical aspiration during sleep. In patients with dysphagia, saliva aspiration has been reported to be more likely in the supine and side-lying positions, whereas pharyngeal retention and laryngeal penetration are absent in the semi-prone position [16]. BMMI is characterized by a disruption of the coughing, breathing, and swallowing centers, resulting in pneumonia, which can be severe. Therefore, positioning should be considered early in the course of the disease to prevent saliva aspiration.

A comprehensive approach to respiratory rehabilitation is essential for treating various symptoms and prolonged respiratory failure caused by BMMI. In addition to physiotherapy, a positional expectorant approach centered on the semi-prone position was implemented every two hours in collaboration with the ward staff. Therefore, further deterioration of respiratory function was prevented, and recurrent AP was avoided. Physical therapists alone cannot adequately respond to patients with severe respiratory symptoms because their intervention time is limited. Therefore, respiratory care should be continued in collaboration with the ward staff.

## Conclusions

Physiotherapy is effective in preventing the exacerbation of various complications caused by BMMI. Prevention of subclinical aspiration, especially during acute physiotherapy, is essential to improve life expectancy. Therefore, early planning of a comprehensive 24-hour multidisciplinary rehabilitation program is essential to prevent worsening complications and functional decline.
